# Trans-UTPA: PSO and MADDPG based multi-UAVs trajectory planning algorithm for emergency communication

**DOI:** 10.3389/fnbot.2022.1076338

**Published:** 2023-01-24

**Authors:** Jie Li, Shuang Cao, Xianjie Liu, Ruiyun Yu, Xingwei Wang

**Affiliations:** ^1^School of Computer Science and Engineering, Northeastern University, Shenyang, Liaoning, China; ^2^School of Software, Northeastern University, Shenyang, Liaoning, China

**Keywords:** multi-UAVs collaboration, PSO, trajectory planning, energy consumption, multi-agent reinforcement learning, transformer

## Abstract

Communication infrastructure is damaged by disasters and it is difficult to support communication services in affected areas. UAVs play an important role in the emergency communication system. Due to the limited airborne energy of a UAV, it is a critical technical issue to effectively design flight routes to complete rescue missions. We fully consider the distribution of the rescue area, the type of mission, and the flight characteristics of the UAV. Firstly, according to the distribution of the crowd, the PSO algorithm is used to cluster the target-POI of the task area, and the neural collaborative filtering algorithm is used to prioritize the target-POI. Then we also design a Trans-UTPA algorithm. Based on MAPPO 's policy network and value function, we introduce transformer model to make Trans-UTPA's policy learning have no action space limitation and can be multi-task parallel, which improves the efficiency and generalization of sample processing. In a three-dimensional space, the UAV selects the emergency task to be performed (data acquisition and networking communication) based on strategic learning of state information (location information, energy consumption information, etc.) and action information (horizontal flight, ascent, and descent), and then designs the UAV flight path based on the maximization of the global value function. The experimental results show that the performance of the Trans-UTPA algorithm is further improved compared with the USCTP algorithm in terms of the success rate of each UAV reaching the target position, the number of collisions, and the average reward of the algorithm. Among them, the average reward of the algorithm exceeds the USCTP algorithm by 13%, and the number of collisions is reduced by 60%. Compared with the heuristic algorithm, it can cover more target-POIs, and has less energy consumption than the heuristic algorithm.

## 1. Introduction

UAVs play an increasingly important role in emergency communication networks. Due to natural disasters, communication infrastructure cannot work properly. Rescue missions need to be fast and agile. The use of multiple UAVs to form an air UAV group self-organizing network can achieve low-latency and high-reliability air-ground coordinated transmission between the UAV group and the ground intelligent terminal equipment (Wang et al., 2021). With the advent of the 6G era, UAVs are expected to offer additional new services such as real-time image transmission, caching and multicast, data dissemination or collection (Wu and Zhang, 2018; Wu et al., 2018) mobile relay and edge computing (Li et al., 2015; Yang et al., 2019; Ma et al., 2021c), and wireless power transmission.

In emergency scenarios, UAV clusters often undertake complex tasks with multiple targets and nodes. The core goal of multi-UAVs task allocation is how to achieve the efficient use of each UAV under the premise of ensuring the completion of the overall task, that is, the overall task allocation balance. The machine learning method enhanced by evolutionary computation (ECML) combines the advantages of ML and EC, and has strong potential. In particular, ECML (Zhang et al., 2022) also has strong search ability, which can greatly reduce the computational cost of cluster node analysis. In the actual construction of UAV cluster task allocation algorithm, genetic algorithm, particle swarm optimization algorithm (Lipare et al., 2021; Krishna et al., 2022; Pu et al., 2022) and ant colony algorithm are often introduced, and bionic intelligence is used to realize better cooperation between UAV cluster individuals, so as to achieve the overall task goal with the best effect (Wangsheng et al., 2021).

Effectively designing the flight path (Jin and Yang, 2021) of UAVs can improve the working efficiency of UAVs. At present, the existing research on single UAV path planning has been very mature. Classical algorithms such as A * algorithm, Dijkstra algorithm, wavefront algorithm and fast exploration random tree algorithm have been widely used in UAVs. With the latest development of machine learning, the research and development of path planning algorithms based on machine learning has been growing rapidly, such as value iterative network, gated neural network and other path planning algorithms. In recent years, with the use of collaborative mission scenarios become widespread, the demand for UAV swarm collaborative path planning (Yao et al., 2016; Jin et al., 2021; Ma et al., 2021a) has become more urgent. The path planning of UAV cluster not only needs to consider the flight distance and energy consumption of single UAV, but also needs to evaluate the safety and cooperation ability of multiple UAVs to ensure that the UAV cluster can perform tasks safely and efficiently. Multi-agent reinforcement learning is the key to solving this problem.

The existing research on multi-agent motion planning problems can be roughly divided into two categories, centralized (Tang et al., 2018) and decentralized methods (Ma et al., 2021b). The centralized method defines the motion planning problem as an optimization problem, where the position, velocity, and target position information of all agents are available. The goal is to guide all agents toward their desired positions, avoid collisions (Tian et al., 2022), and minimize targets (such as energy or time). The decentralized algorithms can be appropriately extended because they allocate the computational effort to multiple agents. They are also very robust to interference when performing real-time calculations.

Although UAVs communication has rich application value, considering the limited airborne energy of a UAV and the need to simultaneously provide energy for propulsion and communication, achieving green UAV communication flight is a key challenge. At present, energy-saving UAVs communication (Liu et al., 2022) can be divided into three categories: (i) given communication requirements, minimize energy consumption; (ii) given total energy/power budget to maximize performance gain; (iii) Maximize energy efficiency. One way to improve energy efficiency is to reduce path loss.

Therefore, we design a PSO-based Trans-UTPA UAVs path planning algorithm. UAV path planning for multi-UAVs cooperative communication and data acquisition is studied. The contributions of this article are as follows:

(1) We propose a PSO-based global optimal target-POI clustering model. We regard each affected person as a particle. Assuming that the particle velocity is constant, we calculate the average distance and standard deviation based on the position of the particle and the position of the cluster center, and minimize the average distance and standard deviation to design the fitness function. All particles change their positions according to the maximum value of the global adaptive function value, so that all particles have clustering centers. We call the population location formed by all particles in a cluster center the target-POI region, where the UAV performs an emergency mission.
(2) We designed a green energy consumption calculation model. The UAV performs flight tasks in a three-dimensional environment, involving flight energy consumption, communication energy consumption and data acquisition energy consumption. When designing multi-UAV cooperative trajectory planning, we comprehensively consider the distance between each UAV and target-POI, the power of each UAV and the priority of target-POI, and select the appropriate UAV to perform flight tasks on target-POI to ensure that all UAVs have low energy consumption while completing flight tasks.
(3) We designed the Trans-UTPA multi-UAV cooperative path planning algorithm. Based on the MAPPO algorithm, we introduce Transformer mechanism to replace the traditional RNN structure for UAVs track sequential modeling. Multi-UAV performs strategy learning based on state information and action information in three-dimensional space, and selects the emergency task with the largest global value function in the evaluation network. The strategy learning of Trans-UTPA has no action space limitation and can be multi-task parallel, which improves the efficiency and generalization of sample processing.
(4) Through the simulation platform, the performance of the algorithm proposed in this paper is tested, and the Trans-UTPA is compared with the USCTP algorithm. The results show that the performance of the algorithm is better than that of the USCTP algorithm in terms of the success rate of the UAV reaching the target, the number of collisions of the UAV and the average reward of the algorithm. Compared with the UAV which executes A * algorithm, it consumes less energy and has certain advantages.

The organization of this chapter is as follows : The second part introduces the existing related research. The third part introduces the clustering recommendation based on PSO. The fourth part introduces the multi-UAVs cooperative path planning constraint model and the multi-UAVs cooperative path planning green energy consumption calculation model and Trans-UTPA algorithm. The fifth part reports the evaluation of the algorithm. We explain the simulation settings, and get the following result diagram through different parameter settings. In the sixth part, a conclusion and the next stage of work are given.

## 2. Related work

In emergency rescue scenarios, the crowds are scattered and the coverage of UAV services is limited. So we define an NP-hard problem: Given N points with a certain distance. We hope to select K cluster centers from the given vertices, and the remaining vertices and cluster centers complete the clustering. Minimize the distance from one vertex to other vertices in a cluster.

In order to solve the NP problem, a large number of clustering algorithms have emerged. The existing clustering methods are usually divided into density-based, hierarchical, graph decomposition and partitioning methods. The density-based algorithm is represented by DB-SCAN (Shinde et al., 2022) CGCA (Kowalski and Jeczmionek, 2022) and other similar methods. Hierarchical clustering methods can use two different strategies : top-down and bottom-up (also known as agglomerative clustering) (Kordos et al., 2022). Finally, the most popular is the partition method using k-means algorithm (Ma et al., 2021d; Sathyamoorthy et al., 2022), although these methods can achieve better clustering effect, because the emergency rescue pay attention to efficient and fast, so we use the PSO on the line clustering, because the PSO algorithm has a local optimal solution and global optimal solution, in order to avoid local convergence, we adjust the particle motion through the global optimal, in addition to PSO aggregation speed is also very fast.

When everyone has their own clustering population, designing a track for the UAV has become an important issue. A good route can improve the efficiency of the UAV. Dynamic path planning in unknown environments has always been a challenge in the current research field (Zhang et al., 2021). The dual Q network (DDQN) deep reinforcement learning proposed by DeepMind is applied to dynamic path planning in unknown environment. A reward-penalty function is set up. With the updating of the neural network and the increase of the probability of greedy rule, the local space searched by an agent is expanded. The results show that the reinforcement learning algorithm enhances the dynamic obstacle avoidance and local planning ability of the agent in the environment. We use multi-agent for multi-UAVs path planning. In addition to meeting obstacle avoidance and networking communication, it can also cover more clustering points and improve the utilization rate of UAV.

In addition, the energy of UAV is limited. How to maximize the efficiency of UAVs under limited energy is an important research content (Ahmed et al., 2016). Three algorithms are proposed to solve the multi-UAVs path planning problem in multiple 2D, barrier-free and discrete planes, while meeting the coverage requirements and minimum energy consumption. Discretize the space and provide a more realistic view of how the UAV travels along its path to ensure a collision-free trajectory. Li W. et al. (2022) proposed an improved probabilistic road map (IPRM) algorithm to solve the energy consumption problem of multi-UAVs path planning. The mathematical model and energy consumption model are established by simulating the real terrain environment. The sampling space of PRM algorithm is optimized to make the path clearer and improve the utilization of space and time.

Zhu et al. (2022) proposed a hexagonal region search (HAS) algorithm, which is combined with multi-agent deep Q network (DQN), called HAS-DQN. By limiting the total coverage of UAVs, HAS-DQN can effectively avoid collision with UAVs. Experiments show that HAS-DQN can effectively solve the path overlapping problem of multiple UAVs moving at the same cost in unknown environment.

In the algorithm design in this paper, we not only consider the energy consumption of UAV flight action, but also consider the energy consumption of UAVs communication and data acquisition. Combining the key degree of POI, the distance from the UAV to the mission area, and the remaining power of the UAV, a reward mechanism is designed to promote the UAV to make the optimal decision, that is, to maximize the global reward.

## 3. Clustering recommendation based on PSO

**Figure 1:**
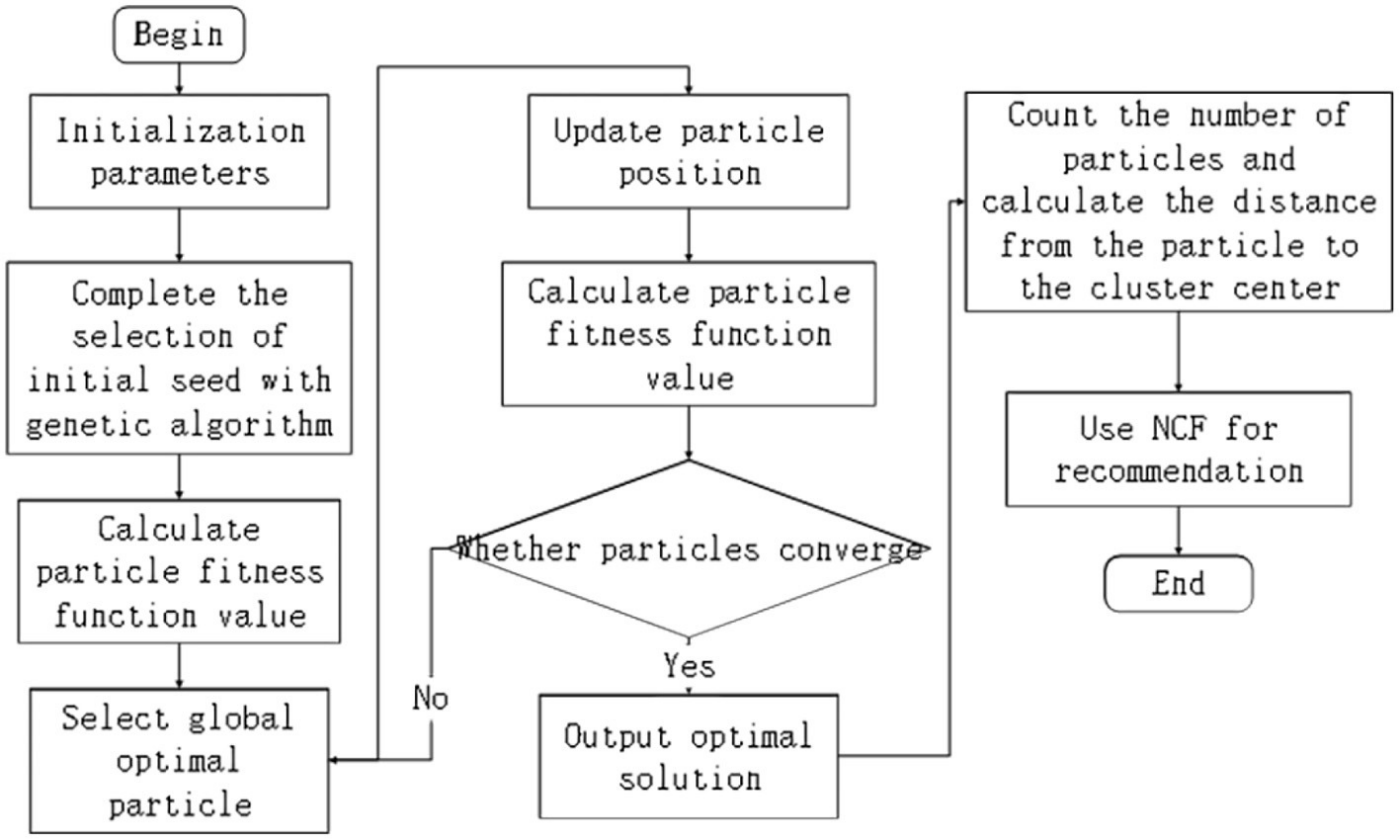
Flowchart of PSO-based clustering recommendation algorithm.

In the emergency communication scenario, the affected population is scattered, and the number of UAVs and endurance energy are limited. In order to enable UAVs to complete emergency rescue missions for emergency areas, we use genetic algorithms to complete the selection of initial seeds. Then, according to the PSO algorithm, the fitness function is calculated according to the location information of the crowd to find the optimal group to complete the crowd clustering, as shown in Figure 1.

### 3.1. Preliminaries

(1) target-POI definition: we define the region formed after clustering as target-POI.
(2) Coverage hypothesis: it is assumed that the size of the area formed after clustering is within the coverage of the UAV.
(3) The PSO speed hypothesis: the speed of the particle motion process is constant, with a constant retrieval speed, recorded as *v*.
(4) UAV to complete the ascending or descending flight energy consumption assumptions:the UAV ascending or descending flight operation energy consumption is constant, the amount of consumption and the number of execution.
(5) UAV data storage: it is assumed that the results of UAV data acquisition can be temporarily stored in UAV memory.

### 3.2. Initial seed selection based on genetic algorithm

The affected people are scattered in the affected area. To complete the clustering, we must first select the clustering center to complete the clustering partition. The selection of cluster centers is particularly important. Correct selection of cluster centers can improve work efficiency and reduce the consumption of some materials and human resources. Genetic algorithms (GAs) are inspired by the theory of nature, which is known for its global adaptability and robust search capabilities to capture good solutions (Sharma and Kaushik, 2021). Therefore, we adopt a genetic algorithm to complete the selection of initial seeds.

First, the chromosome with *K* genes is set to *K* cluster centers, denoted as *X* = {*X*_1_, *X*_2_, *X*_3_, …*X*_*k*_}, where *X*_*k*_ represents an n-dimensional vector, and the other interior points are denoted as *C* = {*C*_1_, *C*_2_, *C*_3_, …, *C*_*i*_}. The latitude and longitude coordinates of *C*_*i*_ is (ψ_1_, α_1_), and the latitude and longitude coordinates of *X*_*k*_ is (ψ_2_, α_2_).During evolution, the fitness function (Equation 1) is used to calculate the sum of the distances (Equation 2) (Wang et al., 2014) from all interior points to the cluster center.


(1) $$\begin{array}{l} Dist\left(C_{i},X_{j}\right)=haversin\left(\frac{d}{R}\right)\\ \;=haversin\left(\psi_{2}-\psi_{1}\right)+cos\left(\psi_{1}\right)cos\left(\psi_{2}\right)haversin\left(\alpha_{2}-\alpha_{1}\right) \end{array}$$

(2) $$\begin{array}{l} F\left(poi\right)=\underset{X_{j}\in X}{\sum }min\left(Dist\left(C_{i},X_{j}\right)\right),1\leq i\leq k \end{array}$$

where *R* represents the radius of the earth, and the value is 6371 km. φ represents the latitude, α represents the longitude.

When the sum of fitness *F*(*poi*) reaches the minimum, the population tends to converge to an optimal chromosome (solution). Once the optimal cluster centers come out, we use them as initial seeds, denoted as *X*_*init*_.

### 3.3. PSO-based clustering recommendation

PSO is a famous bionic optimization algorithm, which is an iterative search algorithm based on population. In PSO algorithm, all particles enter the search space to find the optimal solution. We hope to use the PSO search algorithm to divide the crowd in the rescue area so that the UAV can provide rescue services. In the rescue area, we regard each person as a particle, and design the fitness function according to the average distance and standard deviation between the particle and the cluster center. The next position of each particle is calculated by a fitness function. The larger the global fitness function value is, the particle moves to that position.

After the initial seed is determined, all particles in the clustering region move to the clustering center to complete the clustering operation. Suppose that the cluster region is composed of n particles, denoted by *Y* = {*Y*_1_, *Y*_2_, *Y*_3_, …, *Y*_*n*_}, and the position of the cluster center is denoted by *M* = {*M*_1_, *M*_2_, *M*_3_, …, *M*_*m*_}.

The global optimal value is calculated by the social interaction of the particle in the group, and the best fitness value obtained by the particle in the population. In the search process, a particle not only needs to record its own personal best solution, but also records the overall solution of the cluster center selected by other particles, that is, the shortest reach to the cluster center. We designed a fitness function. The current position of the *i* th particle is denoted as *P*_*i*_, and the average distance from all current particles to the cluster center *M*_*m*_ (Equation 3) and the standard deviation of all particle distances (Equation 4) are calculated.


(3) $$\begin{array}{l} minD=\frac{1}{n-m}\sum ||M_{m}-P_{i}||,i=1,2,3,...,n-m \end{array}$$

(4) $$\begin{array}{l} minC=\sqrt{\frac{\overset{n-m}{\underset{i=1}{\sum }}{\left(D-P_{i}\right)}^{2}}{n-m}} \end{array}$$

Then, the fitness function (Equation 5) for each current particle is designed based on the mean and standard deviation.


(5) $$\begin{array}{l} Fitness=k_{1}D+k_{2}C \end{array}$$

Among them, k1 and k2 represent the weight coefficients between the average distance and the standard deviation, respectively. *Fitness* is the fitness function value.

All particles change their current position according to fitness and make the next action. The action rules are as follows (Equations 6, 7) (Wang et al., 2022):


(6) $$\begin{array}{l} fitness_{i}^{a+1}=fitness_{i}^{a},ifFitness\left(P_{i}^{a+1}\right)\geq Fitness\left(fitness_{i}^{a}\right) \end{array}$$

(7) $$\begin{array}{l} fitness_{i}^{a+1}=P_{i}^{a+1},ifFitness\left(P_{i}^{a+1}\right)<Fitness\left(fitness_{i}^{a}\right) \end{array}$$

where, $P_{i}^{a+1}$ is the next position of the particle, $fitness_{i}^{a}$ represents the current best position of the particle, $fitness_{i}^{a+1}$ represents the new personal best value of the particle, and *a* is the number of iterations. $Fitness\left(P_{i}^{a}\right)$ refers to the fitness function value at the position of $P_{i}^{a}$.


(8) $$\begin{array}{l} globalworth_{i}^{a}=argMin_{i=1Fitness\left(fitness_{i}^{k}\right)}^{n-m} \end{array}$$

Using Equation (8), we can calculate the global optimum, where $globalworth_{i}^{a}$ is the global optimal value of particle *i*. Update your location by ensuring that the globalworth value is larger.

When all particles find their cluster centers, population clustering is completed. Since each particle runs to different cluster centers in the clustering process, the number of particles in each population is different. The number of particles in each population is recorded as *target* − *POI* = {*T*1, *T*2, *T*3, …, *Tm*},. Assuming that the initial release position of the UAV is (*U*_*x*_, *U*_*y*_), the distance from the UAV to the center $\left(T_{x}^{i},T_{y}^{i}\right)$ of the population Ti is calculated to be *D*_*gi*_ (Equation 9).


(9) $$\begin{array}{l} D_{gi}=\sqrt{{\left(U_{x}-T_{x}^{i}\right)}^{2}+{\left(U_{y}-T_{y}^{i}\right)}^{2}} \end{array}$$

Calculate the distance from all particles to the cluster center within population *T*_*i*_, and record the maximum distance as *R*. Then the particle number *N*_*T*_*i*__ and the distance *D*_*gi*_ are converted into sparse vectors, and the neural collaborative filtering algorithm is used to complete the target-POI priority recommendation, indicating which area performs which flight task.

The values in the regional particle number feature *N*_*T*_*i*__ and the distance feature *D*_*gi*_ are converted into sparse vectors, which can be input into the neural network. Then, the input vector is multiplied with the embedding matrix in the embedding layer to obtain the embedded vector representation of the particle number feature and the distance feature. In the fusion layer, the dimension consistency of the particle number feature and the distance feature vector is completed. In the neural collaborative filtering layer, the vectors obtained from the pooling layer capture the nonlinear and high-order correlations between particles and regions through a hidden layer consisting of multiple fully connected layers. Finally, in the prediction layer, the output vector of the last layer is mapped to the final prediction result of target-POI. For target-POI with different priorities, UAVs perform different tasks. Target-POI with the highest priority performs task networking communication tasks, establishes a UAV network, and provides network communication for the crowd. The relatively simple target-POI performs the task of collecting relevant data.

## 4. Multi-UAVs path planning algorithm

### 4.1. Multi-UAVs cooperative path planning constraint model

In the process of emergency rescue, multiple UAVs complete the mission together. Relevant parameter statistics used in the multi-UAVs path planning constraint model are shown in Table 1.

**Table 1:** Statistics of relevant parameters used in multi-UAVs path planning constraint model.

**Parameter**	**Interpretation**
*E*	Onboard energy of a UAV
*T*_*i*_=$\left(T_{i}^{x},T_{i}^{y},T_{i}^{z}\right)$	Location of target-POI i
*O*_*i*_=$\left(O_{i}^{x},O_{i}^{y},O_{i}^{z}\right)$	The location of the i th obstacle
$U_{i}^{t}$=$\left(U_{t}^{Xi},U_{t}^{Yi},U_{t}^{Zi}\right)$	Position of UAV i at time t
*V*_*t*_	UAV flight speed at time t
*Elec*_*t*_	Remaining power of UAV at time t
*E*=$\left\{\left(E_{x}^{i},y,z\right)\cup \left(x,E_{y}^{i},z\right)\cup \left(x,y,E_{z}^{i}\right)|E_{x}^{i}=E_{y}^{i}=E_{z}^{i}=B\right\}$	Rescue area
*B*	B is the boundary value of the rescue area
*V*_*MAX*_	Maximum Flight Speed of UAV
*u*	Number of flying UAVs
*d*_*co*_	Communication distance Upper

Multiple UAVs complete the mission in a three-dimensional environment, need to pay attention to a variety of constraints: (1) Each UAV should fly in the rescue area; Equation (10) (2) In the process of emergency communication, the flight speed Vt of UAV is less than the maximum flight speed of UAV; Equation (11) (3) After the UAV reaches the mission area, it must have enough energy to complete the mission of the target-POI area, and the energy cannot be completely consumed; Equation (12) (4) Multiple access is prohibited in any target-POI zone; Equation (13) (5) Multiple UAVs cannot simultaneously appear in one area. Equation (14), where *i* and *j* denote the i-th and j-th UAVs, respectively;


(10) $$\begin{array}{l} U_{i}^{t}\in E \end{array}$$

(11) $$\begin{array}{l} 0<V_{t}<V_{max} \end{array}$$

(12) $$\begin{array}{l} Elec_{t}>0 \end{array}$$

(13) $$Visit(T_{i})=\{\begin{array}{ll} 1 & \text{Not visited}\\ 0 & \text{visited} \end{array}$$

(14) $$\begin{array}{l} \left(U_{t}^{Xi},U_{t}^{Yi},U_{t}^{Zi}\right)\neq \left(U_{t}^{Xj},U_{t}^{Yj},U_{t}^{Zj}\right) \end{array}$$

The UAV's behavior during maneuvers should be prioritized as it provides smoothness during flight. The flight slope is defined as the maneuverability of the UAV during gliding and climbing. During the flight, the slope of the UAV is moved horizontally from one path point to another (Equation 15).


(15) $$slope_{i}=\{\begin{array}{ll} S & \text{if }slo_{\text{i}}\notin [tan(\alpha_{\text{max}}),tan(\beta_{\text{max}})]\\ 0 & \text{Otherwise} \end{array}$$

where, *slope*_*i*_ is the flying slope from one waypoint to the i-th waypoint; α_*m*_*ax* and β_*m*_*ax* represent the maximum tolerable gliding and climbing angles, and *slo*_*i*_ can be formulated, according to Equation (16) (Mughal et al., 2022), as follows:


(16) $$\begin{array}{l} slo_{i}=\frac{z_{i}-z_{i-1}}{||x_{1}-x_{i-1},y_{i}-y_{i-1}|{|}_{2}} \end{array}$$

where, *slo*_*i*_ is the flying slope taken by the UAV from the i-th waypoint (*x*_*i*_, *y*_*i*_, *z*_*i*_). The UAV flight slope value *slope*_*i*_ complies with constraint (Equation 17).


(17) $$\begin{array}{l} s\leq \frac{1}{2} \end{array}$$

During the flight of the UAV, the maximum safe distance *d*_*safe*_ between the two adjacent UAVs should be guaranteed. The distance between the UAV *i* and the UAV *j* is calculated to be *D*_*ij*_ using Equation (18). When calculating the path of multiple UAVs, it is necessary to ensure that the UAVs are not too close. In order to maintain the safe distance between them, the limit can be expressed as follows (Equation 19):


(18) $$\begin{array}{l} D_{ij}=\sqrt{{\left(U_{t}^{Xi}-U_{t}^{Xj}\right)}^{2}+{\left(U_{t}^{Yi}-U_{t}^{Yj}\right)}^{2}+{\left(U_{t}^{Zi}-U_{t}^{Zj}\right)}^{2}} \end{array}$$

(19) $$\begin{array}{l} D_{ij}>d_{safe} \end{array}$$

In addition, the UAV should also pay attention to the distance between the obstacle (Equation 20) to avoid collision between the UAV and the obstacle, so set the flight constraint between the UAV and the obstacle (Equation 21).


(20) $$\begin{array}{l} D_{obs}=\sqrt{{\left(U_{t}^{Xi}-O_{x}^{i}\right)}^{2}+{\left(U_{t}^{Yi}-O_{y}^{j}\right)}^{2}+{\left(U_{t}^{Zi}-O_{z}^{i}\right)}^{2}} \end{array}$$

(21) $$\begin{array}{l} D_{obs}>d_{safe} \end{array}$$

### 4.2. Green energy consumption calculation model for multi-unmanned cooperative path planning

Green energy consumption requires UAVs to consume the least energy and complete more missions during flight. We study the four basic operations of UAV flight operations: horizontal flight, ascent, descent, and hover. The horizontal flight energy consumption of UAV *i* at time *t* is recorded as $E_{level}^{i}\left(t\right)$, and the horizontal energy consumption calculation formula is Equation (22). The energy consumption of the UAV to complete a rise or fall is recorded as *E*_*u*_ and *E*_*d*_, respectively.


(22) $$\begin{array}{l} E_{level}^{i}\left(t\right)=\sigma \left(c_{1}||v_{t}^{i}|{|}^{3}+\frac{c_{2}}{\left||v_{t}^{i}|\right|}\right)\;i=1,2,3,...,n \end{array}$$

where σ represents the slot length, *c*_1_ and *c*_2_ are constants.

The flight operation of the UAV also involves hovering operation. We record the hovering time as *t*_*hover*_, and the hovering energy consumed by the ith UAV in 1 min is recorded as $E_{hover}^{i}\left(t\right)$. Therefore, the calculation of the energy consumed by the i UAV in performing flight operations (Equation 23) is recorded as $E_{fly}^{i}$.


(23) $$\begin{array}{l} E_{fly}^{i}\left(t\right)=E_{level}^{i}\left(t\right)T_{fly}+P_{up}*E_{up}^{i}+P_{down}*E_{down}^{i}+t_{hover}E_{hover}^{i}\left(t\right) \end{array}$$

The time consumed during the flight (excluding hover) is recorded as *T*_*fly*_,*P*_*up*_ is the number of rising, *P*_*down*_ is the number of falling.

In the multi-UAVs mission, if the distance between the UAVs is less than the maximum distance *d*_*com*_ that can be communicated between the UAVs, the UAVs communicate with each other to exchange information, and finally summarize it to a UAV. The information exchanged includes: the amount of existing energy, target-POI access, etc. In this article, we use the location with the highest demand as the network communication area, while other areas are engaged in data collection.

The UAV flight mission includes data acquisition and networking communication. So we have to calculate the UAV data acquisition energy consumption and network communication energy consumption. We refer to the location-critical communication model proposed in Qin et al. (2018). When the UAV accesses target-POI, the access information is transmitted to the nearby UAV, considering the energy consumption $E_{tran}^{ij}$ of one transmission between UAV i and UAV j. The calculation formula (Equation 24) is as follows:


(24) $$\begin{array}{l} E_{tran}^{ij}=Bit*{\left(D_{ij}\right)}^{\alpha }*E_{m}^{B} \end{array}$$

Among them, *Bit* represents the size of the transmission information, $E_{m}^{B}$ represents the energy consumed by transmitting 1 Mbit information within 1 km distance, and α represents the transmission loss index of the transmission medium.

In the three-dimensional environment, the UAV needs to rotate to complete the task of data acquisition. The rotation angle is also a factor that determines energy consumption. We use (Fu et al., 2018) to calculate the energy consumption caused by the rotation angle during the acquisition process as *E*_*corner*_, and the formula is Equation (25):


(25) $$\begin{array}{l} E_{corner}=\overset{n}{\underset{i=1}{\sum }}w_{n}*Q \end{array}$$

where *w*_*n*_ represents the angle of rotation of the nth target-POI UAV, and *Q* represents the energy consumed by one rotation. So UAV i access n target-POIs to complete the rotation of the fuselage and data collection tasks consumed energy recorded as $E_{collect}^{i}$, the formula is as follows (Equations 26, 27):


(26) $$\begin{array}{l} E_{collect}^{i}=P_{collect}*T_{target-POI}^{j}*n+E_{corner} \end{array}$$

(27) $$\begin{array}{l} T_{target-POI}^{j}=\int_{0}^{2R_{max}}\frac{1}{V_{t}}\text{d}r \end{array}$$

After completing n target-POI missions in flight time *T*_*fly*_, the residual energy of UAV i is calculated by Equation (28).


(28) $$\begin{array}{l} Elec_{t}=E-E_{fly}^{i}-\overset{u-1}{\underset{i=0}{\sum }}\overset{u}{\underset{j=1}{\sum }}E_{tran}^{ij}-E_{collect}^{i} \end{array}$$

### 4.3. Trans-UTPA algorithm

#### 4.3.1. Trans-UTPA algorithm framework

**Figure 2:**
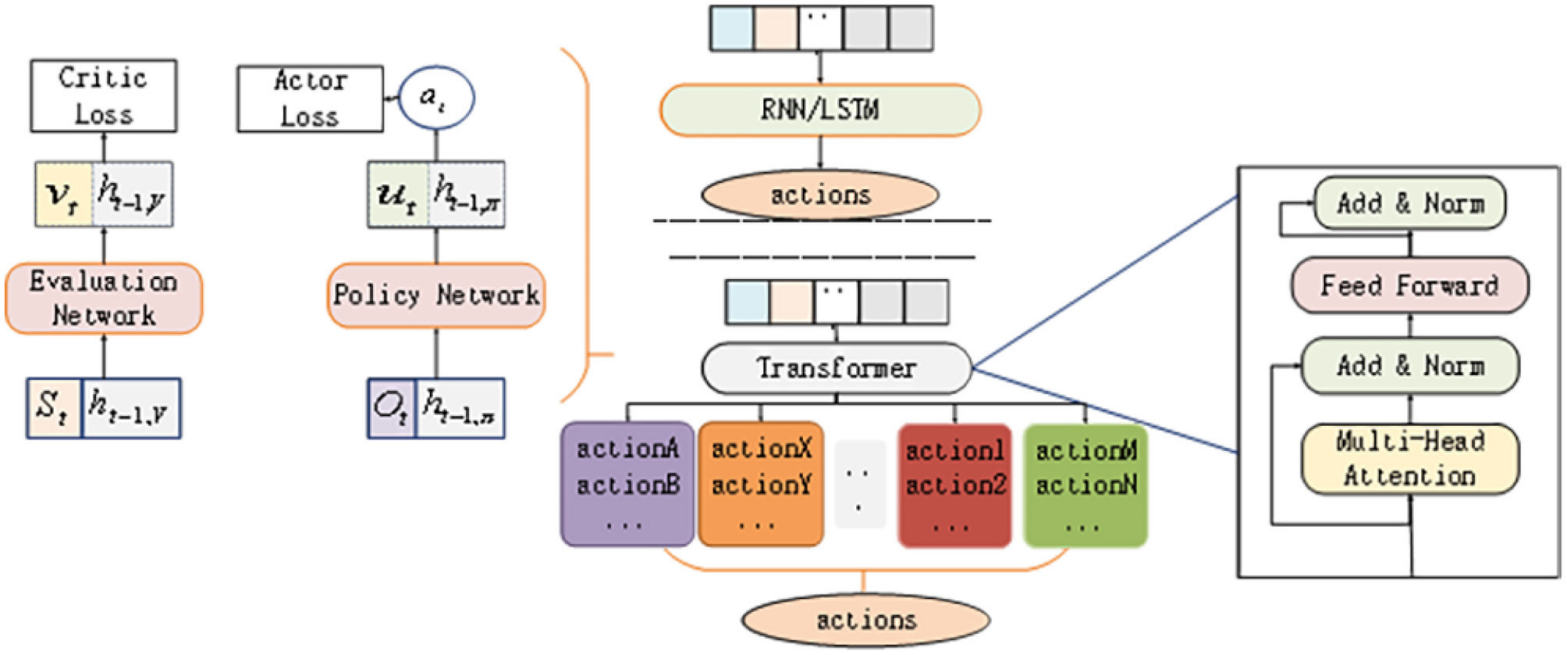
Trans-UTPA algorithm framework.

The architecture of the whole algorithm is shown in Figure 2. The individual value function of each UAV can be calculated using the self-attention mechanism. Since Trans-UTPA is composed of two independent networks, namely the policy network π_θ_(*a*_*i*_|*o*_*i*_) containing parameter θ and the evaluation network *V*_ϕ_(*s*) containing parameter ϕ, these two networks are processed separately. All UAVs share the critic network, and each UAV has its own actor-critc network. First, the collected trajectory is recorded as τ = τ_1_, τ_2_, …, τ_*n*_,*n* represents the number of UAVs, and the trajectory of a single UAV *i* is recorded as $\tau_{i}=\left\{s_{i}^{\left(t\right)},o_{i}^{\left(t\right)},a_{i}^{\left(t\right)}\right\}$.

#### 4.3.2. Design of strategy function and value function based on Transformer

The state information $S_{i,1}^{\left(t\right)},..,S_{i,m}^{\left(t\right)}$ of each UAV at time *t* is input into an embedded layer for representation, and the state value $S_{i}^{\left(t\right)}$ of the UAV in the environment at time *t* is obtained (Equations 29, 30):


(29) $$\begin{array}{l} O_{i}^{\left(t\right)}=E\left(o_{i,1}^{\left(t\right)}\right),E\left(o_{i,2}^{\left(t\right)}\right),...,E\left(o_{i,m}^{\left(t\right)}\right) \end{array}$$

(30) $$\begin{array}{l} S_{i}^{\left(t\right)}=E\left(s_{i,1}^{\left(t\right)}\right),E\left(s_{i,2}^{\left(t\right)}\right),...,E\left(s_{i,m}^{\left(t\right)}\right) \end{array}$$

**Figure 3:**
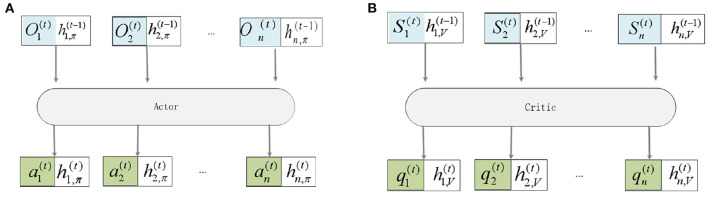
Embedded representation of observations. **(A)** This is Actor framework. **(B)** This is Critic framework.

The predicted action and value of the UAV at time t are Equations (31), (32):


(31) $$\begin{array}{l} h_{i,\pi }^{\left(t\right)},a_{i}^{\left(t\right)}=\pi \left(O_{i}^{\left(t\right)},h_{i,\pi }^{\left(t-1\right)},\theta_{i}\right) \end{array}$$

(32) $$\begin{array}{l} h_{i,V}^{\left(t\right)},q_{i}^{\left(t\right)}=V_{i}\left(S_{i}^{\left(t\right)},h_{i,V}^{\left(t-1\right)},\phi_{i}\right) \end{array}$$

where $h_{i,\pi }^{\left(t-1\right)}$ represents the state of the hidden layer *t* at a time on the actor, $h_{i,V}^{\left(t-1\right)}$ represents the state of the hidden layer at a time on the critic, $O_{i}^{\left(t\right)}$ represents the observation embedding value of the UAV *i* to other UAVs, $S_{i}^{\left(t\right)}$ represents the state embedding value of each UAV, θ_*i*_ is the parameter defining π, and ϕ_*i*_ is the parameter defining *V*_*i*_. In this way, the critic network of UAV *i* evaluates the state of other UAVs.As shown in Figure 3.

Here the hidden layer is treated as part of each UAV, each input holds its own hidden layer, and each output maps to a new hidden layer for the next time step. Using *n* to represent the number of UAVs, *L* to represent the number of fully connected layers, the actor network input and output relationship expression are Equations (33), (34):


(33) $$\begin{array}{l} R_{\pi }^{1}=\left\{h_{1,\pi }^{\left(t-1\right)},h_{2,\pi }^{\left(t-1\right)},...,h_{n,\pi }^{\left(t-1\right)},O_{i}^{\left(t\right)}\right\} \end{array}$$

(34) $$\begin{array}{l} \left\{h_{1,\pi }^{\left(t\right)},h_{2,\pi }^{\left(t\right)},...,h_{n,\pi }^{\left(t\right)},O_{L}^{\left(t\right)}\right\}=R_{\pi }^{L} \end{array}$$

The input-output relationship expression of critic network are Equations (35), (36):


(35) $$\begin{array}{l} R_{V}^{1}=\left\{h_{1,V}^{\left(t-1\right)},h_{2,V}^{\left(t-1\right)},...,h_{n,V}^{\left(t-1\right)},S_{1}^{\left(t\right)}\right\} \end{array}$$

(36) $$\begin{array}{l} \left\{h_{1,V}^{\left(t\right)},h_{2,V}^{\left(t\right)},...,h_{n,V}^{\left(t\right)},S_{L}^{\left(t\right)}\right\}=R_{V}^{L} \end{array}$$

Because of the centralized training distributed execution mode, it is necessary to calculate the global value function, and the global function can be calculated by the individual value function. The global action value function can be calculated using all individual action value functions. The formula is as follows (Equation 37):


(37) $$\begin{array}{l} V_{\pi }\left(s\right)=f\left(q_{i}^{t},...,q_{n}^{t}\right) \end{array}$$

where *f* denotes the credit allocation function for each UAV. In this study, the calculation method in Value-Decomposition Networks (VDN) is used, and the summation function is used to solve the credit allocation problem of UAVs. The calculation formula is shown in Equation (38):


(38) $$\begin{array}{l} f\left(q_{i}^{t},...,q_{n}^{t}\right)=\overset{n}{\underset{i=1}{\sum }}\left(q_{i}^{t}\right) \end{array}$$

#### 4.3.3. Transformer internal attention calculation

The Transformer in the traditional sequence modeling task is input by position coding, and the hidden representation of the coding is automatically regressed and decoded. This article uses a mechanism with a lower triangular matrix to calculate attention (Equations 39–42):


(39) $$\begin{array}{l} Attention\left(Q,K,V\right)=softmax\left(\frac{QK^{T}}{\sqrt{d_{k}}}+M\right)V \end{array}$$

(40) $$\begin{array}{l} R_{i}^{1}=\left\{h_{i}^{\left(t-1\right)},O_{i}^{\left(t\right)}\right\} \end{array}$$

(41) $$\begin{array}{l} Q_{i}^{k},K_{i}^{k},V_{i}^{k}=L_{Q,K,V}\left(R_{i}^{k}\right) \end{array}$$

(42) $$\begin{array}{l} R_{i}^{\left(k+1\right)}=Attention\left(Q_{i}^{k},K_{i}^{k},V_{i}^{k}\right) \end{array}$$

where *M* is the mask matrix, which ensures that the input at time step *t* can only be associated with the input from < 1, 2, …, *t* − 1 >. Where *K, Q, V* represent keys, queries, values respectively. *d*_*k*_ represents a scaling influence factor equal to the key dimension.


(43) $$\begin{array}{l} V_{i}\left(S_{i}^{\left(t\right)},h_{i,V}^{\left(t-1\right)},\phi_{i}\right)=P\left(R_{i}^{L},u_{i}^{\left(t\right)}\right) \end{array}$$

where *L* represents a linear function used to calculate *Q, K, V*. The output $R_{i}^{L}$ of the last Transformer layer is mapped to the space of *V*_*i*_, using a linear function *P* to implement the prediction, as shown in Equation (43):

#### 4.3.4. Algorithm description

The Trans-UTPA algorithm is based on the MAPPO algorithm and is also composed of two independent networks, namely, the policy network π_θ_(*a*_*i*_|*o*_*i*_) with the parameter θ and the evaluation network *V*_ϕ_(*s*) with the parameter ϕ. First collect trajectory τ = {τ_1_, τ_2_, …, τ_*n*_} where UAV *i* ' s trajectory $\tau_{i}=\left\{s_{i}^{\left(t\right)},o_{i}^{\left(t\right)},a_{i}^{\left(t\right)}\right\}$, *t* ∈ (1, *T*), *T* is the training duration.

Initialize the hidden layer state of actor and critic, input the observation value into the actor network to obtain the optional action, and input the state value into the critic network to obtain the reward value. Perform action *a*_*t*_, observe *r*_*t*_,*s*_*t*+1_,*o*_*t*+1_, then write it into τ_*i*_, and calculate the dominance function Â through GAE. The calculation formula is shown in Equation (44):


(44) $$\begin{array}{l} Â\left(s,a_{i}\right)=\underset{t}{\sum }\gamma^{t}r\left(s,a_{i}\right)-V_{\phi }\left(s\right) \end{array}$$

The calculation formula of importance weight is as follows (Equation 45):


(45) $$\begin{array}{l} \omega =\pi_{\theta }\left(o_{i},a_{i}\right)/\pi_{\theta old}\left(O_{i},a_{i}\right) \end{array}$$

Update the actor network parameter θ_*i*_ of UAV *i* (Equation 46):


(46) $$\theta_{i}=argmaxE_{t}[min(\omega \hat{A_{t}},clip(\omega ,1-\varepsilon ,1+\varepsilon )\hat{A_{t}}]$$

Update the parameter ϕ of the critic network of the UAV (Equation 47):


(47) $$\phi =argmin\frac{1}{2}[\sum_{t}(\gamma^{t}r(s,a_{i})-V_{\phi }(s){]}^{2}$$

Based on the above content, the specific UAV swarm cooperative path planning algorithm based on Trans-UTPA is shown in Algorithm 1.

**Algorithm 1:** Cooperative path planning algorithm for UAV swarm based on Trans-UTPA (TUTPA).

**BEGIN**
Initialize UAV parameters, power consumption parameters, action space dimension *action*_*dim* and observation space dimension *obs*_*dim*;
Initialize training parameters Parameter θ of actor network π(*a*|*o*),ϕ parameter of critic network *V*(*s*), learning rate α, discount factor γ, number of training rounds;
Initializes the hidden-layer state *h*_π_ of the actor, the hidden-layer state *h*_*V*_ of the critic;
for *i* = 1, 2, 3, …, *episodes* do:
Initialize data buffer *D* = {}
for *j* = 1 to batch_size *do*:
Set empty list *l* = []
The observation value is input into the actor network to get the optional action *a*_*t*_, and the state value is input into the critic network to get the value v.
Execute action *a*_*t*_ to get reward value *r*_*t*_, observation value *o*_*t*+1_ for the next moment, global state value *s*_*t*+1_;
Write the resulting value *a*_*t*_, *r*_*t*_, *o*_*t*+1_, *s*_*t*+1_ to the list *l*
According to *l*, calculating Dominance Function Â by Formula $A\left(s,a_{i}\right)=\underset{t}{\sum }\left(\gamma^{t}\right)r\left(s,a_{i}\right)-V_{\phi }\left(s\right)$;
Write *l* into buffer *D*;
end for;
Random sampling mini-batch *H* from *D*;
Calculate the loss function *policy*_*loss* of the actor from the data in H and the loss function *value*_*loss* of the critic;
Update parameter θ_*i*_of actor network of UAV *i*, update parameter ϕ of critic network
Select random mini-batch as *b* from *B*
end for
**END**

## 5. Experiment

### 5.1. PSO-based clustering

**Figure 4:**
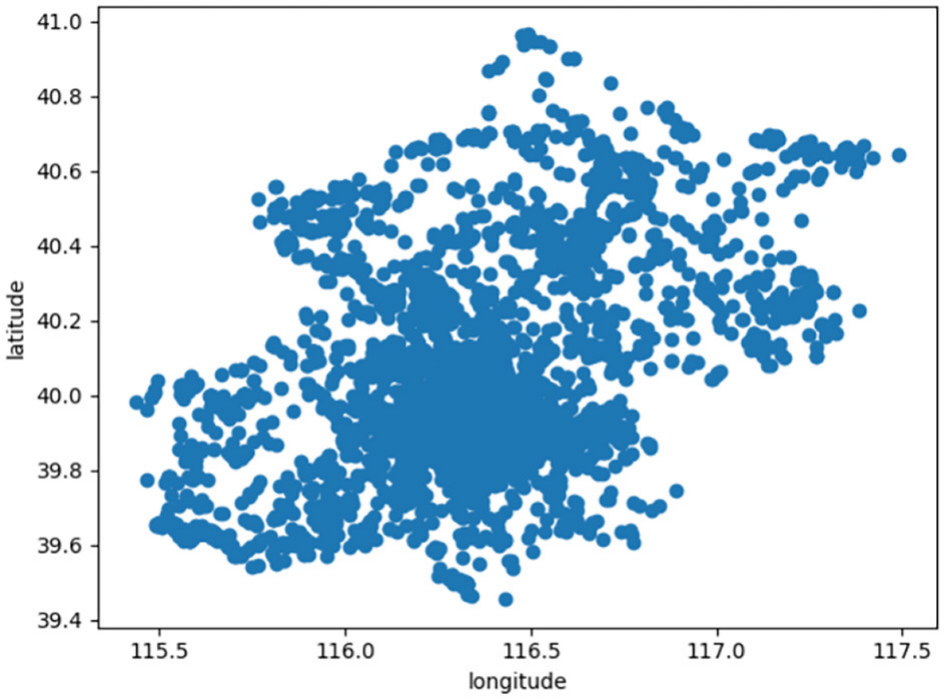
It shows the location distribution of scenic spots in Liaoning.

The data set used in this paper is 7,366 scenic spot location information obtained from Mafengwo Tourism Online, which is represented in the coordinate map. As shown in Figure 4, the abscissa represents the longitude value, and the ordinate is the dimension value.

Each location information in the dataset includes attributes: longitude, dimension, and label (region attribution). The location information of the attractions is shown in Table 2.

**Table 2:** Sample location information for attractions.

**Longitude**	**Latitude**	**Region ownership**
116.47545	40.96251398	Beijing
116.4757993	40.96299956	Beijing
116.4862425	40.96156428	Beijing
123.46856	41.808196	Shenyang
123.407483	41.799246	Shenyang
123.362155	41.786272	Shenyang

**Figure 5:**
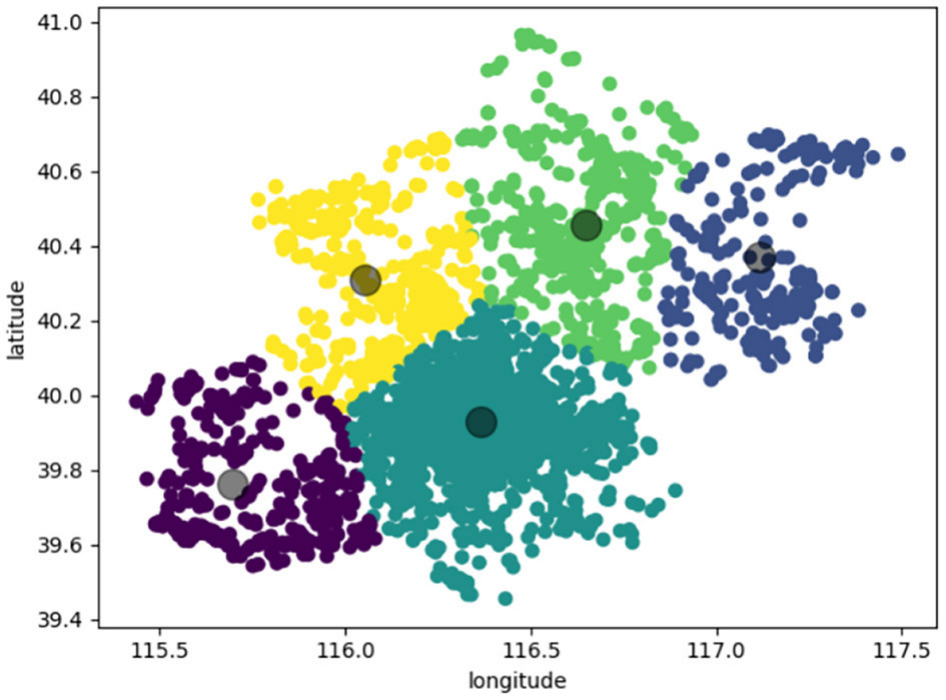
PSO algorithm attractions clustering distribution map.

In the experiment, we use PSO algorithm to cluster the obtained location information. Here *K* = 5, after 10,000 iterations, 1,666 attractions are clustered into 5 clusters. The results are shown in Figure 5. The circles of different colors are used to represent the attractions belonging to different clusters, and the large black circle represents the cluster center.

### 5.2. target-POI recommendation based on PSO

#### 5.2.1. Acquisition of experimental data

In the emergency rescue scenario, it is necessary to comprehensively consider the distance between the rescue crowd and the UAV, the crowd density and the rescue time in the rescue area, and reasonably dispatch the UAV to complete the emergency rescue task. In this experiment, the scenic spot data set in the Mafengwo tourism network is used. The target-POI position is represented by the location information of the scenic spot, and the number of visits represents the crowd density of the position. The attributes of each attraction include: the number of visits to the attraction, the location of the attraction (latitude and longitude), the name of the attraction, and the ID of the attraction. Access statistics based on attractions information are shown in Table 3.

**Table 3:** Sample visit statistics based on attraction information.

**ID**	**Name**	**Location**	**Visits**	**The latest statistical time**
1	Shenyang expo park	41.863424, 123.645846	2,999	May 2022
2	Guanmenshan national forest park	41.121535, 124.188346	1,355	March 2022
3	Benxi water cave	41.304582, 124.08402	1,256	June 2022
4	Shenyang imperial palace	41.803551, 123.462138	1,000	October 2022

#### 5.2.2. Evaluation criteria

In order to evaluate the performance of the algorithm for target-POI recommendation, we use Normalized Discounted Cumulative Gain (NDCG) and Hit Ratio (HR) to evaluate the performance of the algorithm in top-K recommendation.

The calculation formula of HR is shown in Equation (48). *hits*(*i*) records the predicted score of the sample, whether it is in the recommended first *K*, is 1, otherwise it is 0, and *N* represents the total number of samples in the test set.


(48) $$\begin{array}{l} HR=\frac{\overset{N}{\underset{i=0}{\sum }}\left(hits\left(i\right)\right)}{N} \end{array}$$

NDCG is a measure of ranking, which tends to evaluate the order of recommendation. The calculation formula is shown in Equation (49).


(49) $$\begin{array}{l} NDCG_{K}=\frac{DCG_{K}}{IDCG_{K}} \end{array}$$

(50) $$\begin{array}{l} DCG_{K}=\overset{K}{\underset{i=0}{\sum }}\left(\frac{y_{i}}{\log_{2}^{i+1}}\right) \end{array}$$

The DCG obtained by the Equation (50) represents the cumulative gain of the loss, in which the ranking order factor will be considered. Starting from the first item in the obtained ranking, each item is multiplied by the decreasing coefficient, so that the top item gain is higher, and the following items will have a loss.

#### 5.2.3. Analysis of experimental results

**Figure 6:**
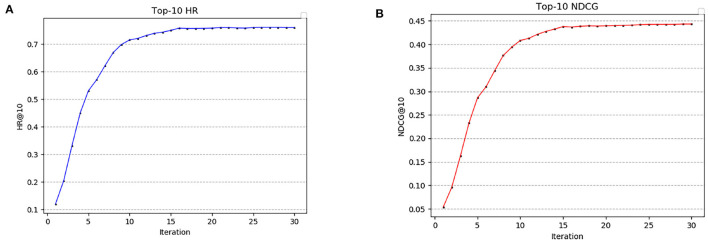
Hit rate and normalized cumulative gain of top-10. **(A)** Top-10 HR. **(B)** Top-10 NDCG.

Outputs the heat ranking of attractions in the area based on the area 's location data and number of visits to the attraction. Figure 6 shows the changes of HR and NDCG in each iteration of the model when taking top-10 recommendation, and the total number of iterations of the experiment is 30. It can be seen from Figure 6A that the growth rate of the model is obvious in the first 10 iterations. When iterating to 15 times, the model begins to converge and HR is 0.7347. When iterating to the 25 th time, the fluctuation range of HR is small and stable in the range of [0.7586, 0.7607]. As can be seen from Figure 6B, NDCG also changes greatly at the initial stage of iteration. When iterating to 15 times, the NDCG is 0.4379. When iterating to 25 times, the NDCG fluctuates between [0.4422, 0.4434].

**Figure 7:**
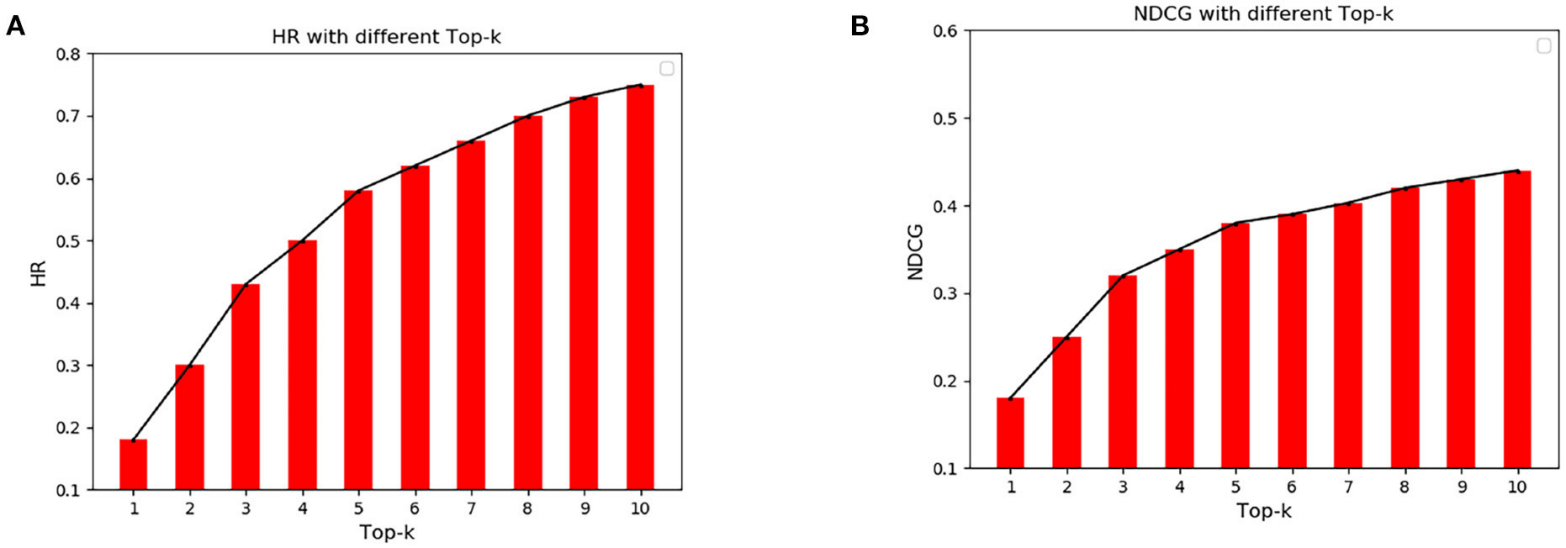
Cumulative Gain of Hit Rate and Normalized Discount at different top-K. **(A)** Hit rate under different top-k. **(B)** Cumulative increase of normalized loss for different top-k.

Figure 7 shows the numerical comparison of HR and NDCG at different top-K recommendations. It can be seen from Figure 7A that HR increases greatly before is <3, and then the HR gap between top-K gradually narrows with the increase of *K*. HR fluctuates around 0.75. Similarly, it can be seen from Figure 7B that the NDCG increases greatly before is <3, and then the NDCG gap between top-K gradually narrows with the increase of. When, NDCG fluctuates around 0.44.

From the results in Figures 6, 7, it can be seen that the HR and NDCG values are higher when using the algorithm for top-K recommendation. When *K* is 10, HR can reach 0.68 and NDCG can reach 0.45. It shows that the algorithm can be used to predict the popularity of scenic spots and get the target-POI ranking that UAVs need to prioritize.

### 5.3. Experiment of UAV swarm cooperative path planning algorithm based on Trans-UTPA

#### 5.3.1. Experimental environment

**Figure 8:**
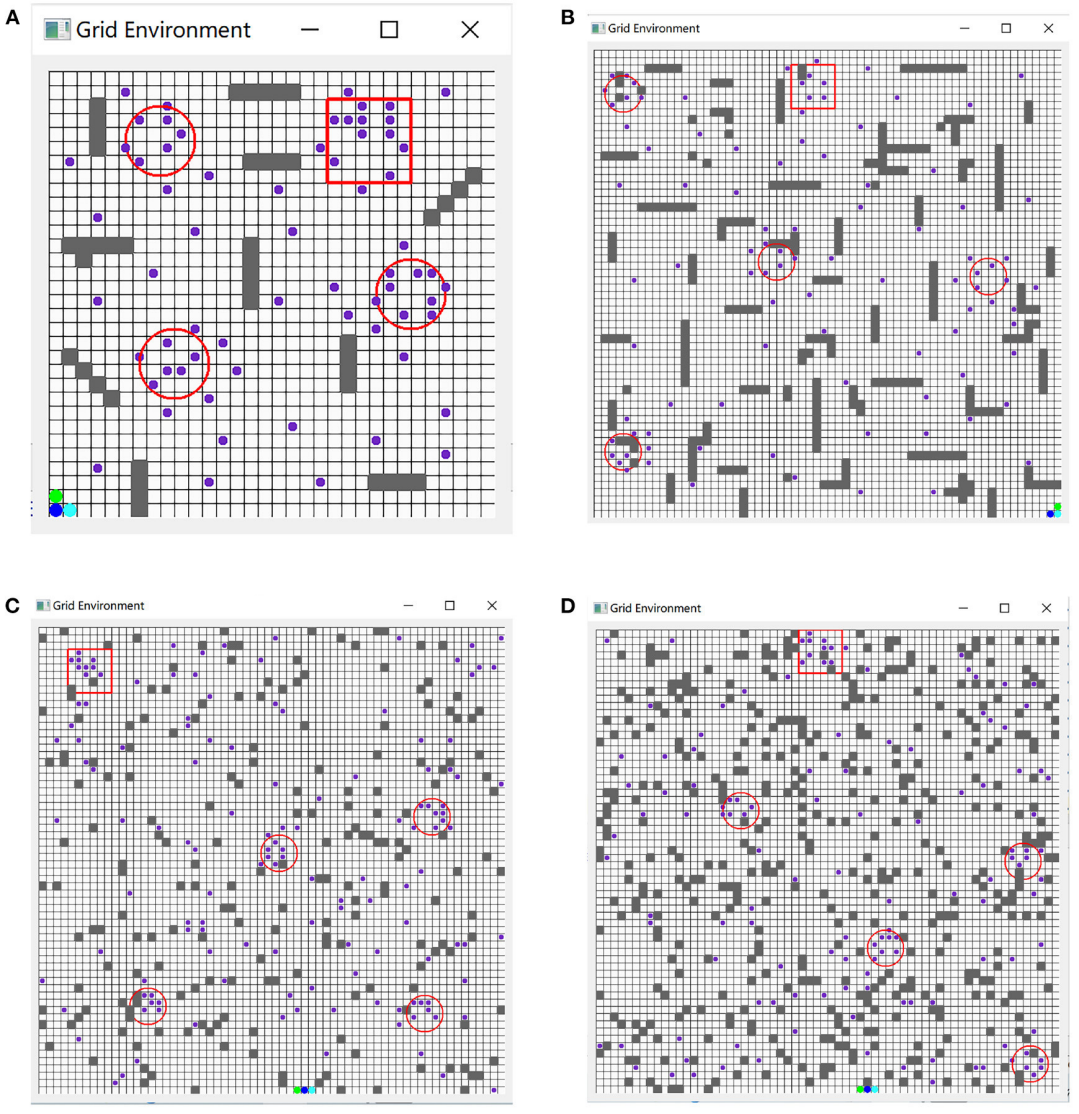
Scene maps of different specifications. **(A)** 32*32 map of fixed scene. **(B)** 64*64 map of fixed scene. **(C)** 64*64 map with a random scene with obstacle density = 0.1. **(D)** 64*64 map with a random scene with obstacle density = 0.3.

The experimental environment of this algorithm includes a training environment and a test environment. The test environment is a given map_ size, the number of UAVs, the number of TaskPOIs, the starting position of the UAV, and the generation density of obstacles to generate obstacles. Randomly distributed test scenarios based on global environmental data. The purple dots are clustered to obtain the target-POI area marked by a red circle. The square red area is the target area for all UAVs to finally network. The following is the environmental scene map of the unreleased UAV after clustering. Figure 8 shows the training environment under different specifications, where Figures 8A, B are the environment maps of 32*32 and 64*64 under fixed scenarios, respectively. Figure 8C shows a 64*64 environment map with an obstacle generation density of 0.1. Figure 8D shows a 64 * 64 environment map with an obstacle generation density of 0.3. Obstacles in the environment are represented by gray squares, and green and blue circles represent UAVs. In order to ensure that the scenes used by various algorithms are consistent, the scene files used by the USCTP algorithm (Li J. et al., 2022) are saved to generate the corresponding fixed scenes, and then compared with the Trans-UTPA algorithm.

#### 5.3.2. Evaluation criteria

We mainly consider the success rate of the UAV reaching the target position, the number of collisions during the movement and the average reward of the algorithm.

(1) Success rate: The probability of each UAV reaching the target position accurately is counted, that is, whether they have completed the task of reaching the target position within a given time or number of times.
(2) The number of collisions: the action conflict between dynamic agents and the conflict between agents and static obstacles, that is, during the movement of the UAV, the number of collisions between the UAV and other UAVs in an episode or between the UAV and obstacles in the environment is counted.
(3) Average reward: each UAV relies on obstacle avoidance during flight, performs access tasks, and controls power consumption to obtain corresponding rewards and penalties. After training several times, the average reward of the UAV reaches a convergent state, indicating that the UAV has trained a rough trajectory and will not be blindly explored again.

#### 5.3.3. Analysis of experimental results

**Figure 9:**
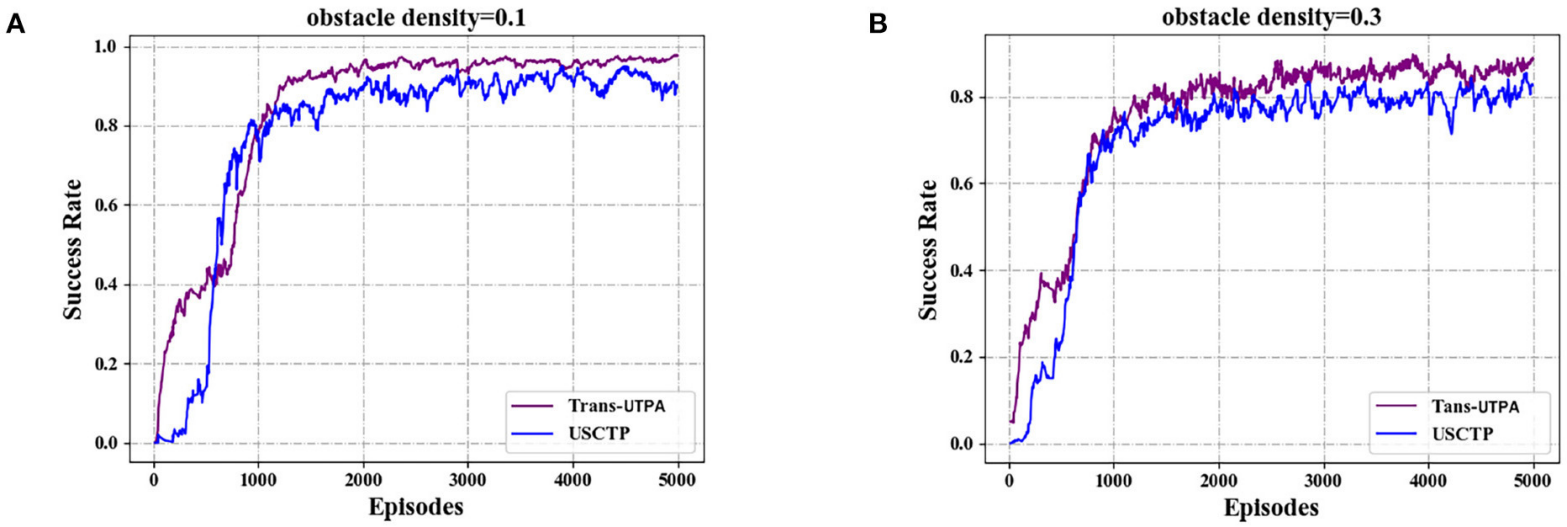
Comparison of success rates of UAV swarms reaching target locations. **(A)** Success rate with obstacle density = 0.1. **(B)** Success rate with obstacle density = 0.3.

The model compares the success rate of reaching the target position, the number of collisions and the average reward of the two algorithms in the test environment 32 * 32 map with different obstacle densities. Figure 9 shows the comparison of the success rate of the UAV swarm based on the two algorithms to reach the target position under different obstacle densities. Figure 9A is the success rate of the UAV reaching the target position when the density is 0.1, and Figure 9B is the success rate of the UAV reaching the target position when the density is 0.3. It can be seen from the figure that under the Trans-UTPA algorithm, the success rate of the UAV swarm to reach the target position has always been higher than that of the USCTP algorithm. With the increase of the number of iterations, the more than part reaches about 8%.

**Figure 10:**
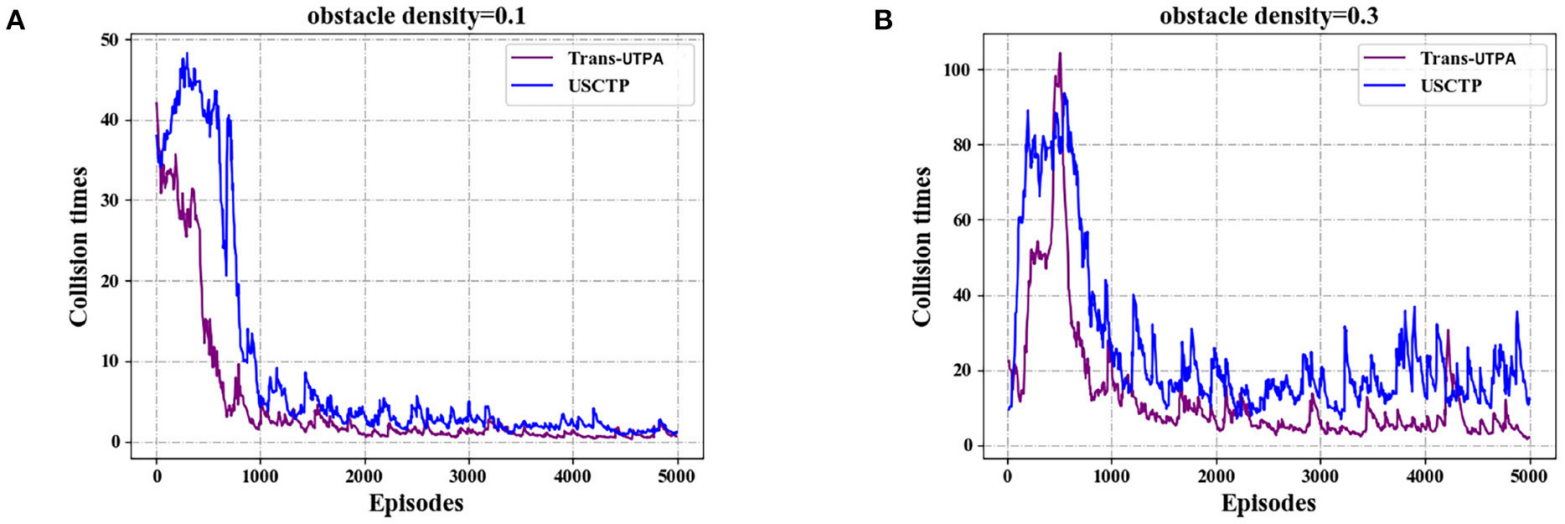
Comparison experiment of collision times of UAVs. **(A)** Collision times with obstacle density = 0.1. **(B)** Collision times with obstacle density = 0.3.

Figure 10 shows the number of collisions of the two algorithms in the flight process of the UAV under different obstacle densities. Figure 10A is the number of collisions of the UAV in the case of a density of 0.1. It can be seen from the graph that the number of collisions of the Trans-UTPA algorithm has been lower than the USCTP algorithm. Even in the 300–400 rounds, the peak is still lower than the USCTP algorithm, and the convergence speed is faster than the USCTP algorithm. Figure 10B shows the number of collisions of UAVs at a density of 0.3. Compared with the case of obstacle density of 0.1, the number of collisions of UAVs under the two algorithms is significantly increased, but it can be seen from the figure that the number of collisions of UAVs based on Trans-UTPA algorithm is the lowest.

**Figure 11:**
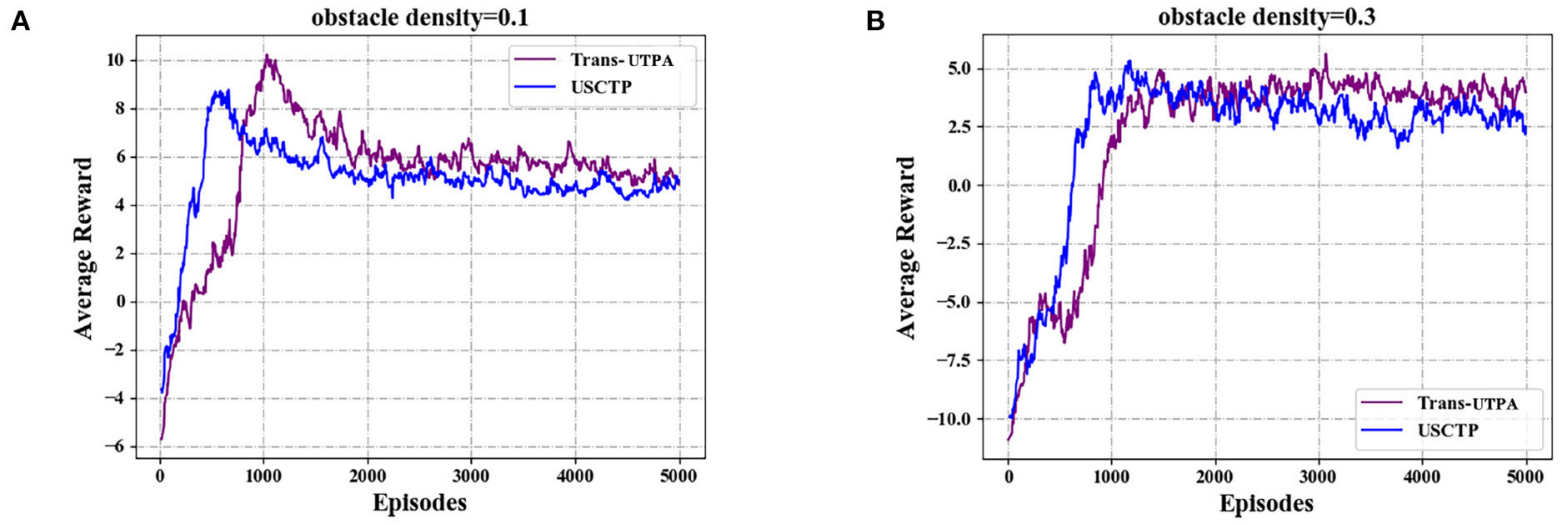
Comparison experiment of the average rewards. **(A)** Average rewards with obstacle density = 0.1. **(B)** Average rewards with obstacle density = 0.3.

Figure 11 shows the average rewards obtained by the UAV swarms of the two algorithms during flight under different obstacle densities. Figure 11A is the average reward value of the two algorithms under the density of 0.1. It can be seen from the figure that the average reward value based on the Trans-UTPA algorithm has always been higher than the USCTP algorithm. Figure 11B shows the average reward value of the two algorithms when the density is 0.3. It can be seen from the figure that the two algorithms are not much different. In the first 1,000 rounds, the USCTP algorithm is higher. As the number of rounds increases, Trans-UTPA is gradually higher than the USCTP algorithm.

#### 5.3.4. Energy consumption experimental results

In the energy consumption part, we use simulation experiments to verify the Trans-UTPA algorithm. The relevant parameters in the experiment are shown in Table 4.

**Table 4:** Statistics of UAV simulation parameters.

**Parameter**	**Value**
*E*	5.3*10^4^*J*
*d*_*safe*_	400*m*
*c*_1_, *c*_2_	0.0661, 15.97
σ	0.2
*Bit*	1*Mbit*
*V*_*MAX*_	10*m*/*s*
*u*	3
α	2
$E_{m}^{B}$	1*J*/(*m*^α^.*Mbit*)
*P*_*collect*_	0.37*J*/*s*
$E_{hover}^{i}\left(t\right)$	10*J*/*min*
*Q*	10*J*/*r*
*d*_*com*_	20*m*
$E_{up}^{i}$	2.5*Jeachtime*
$E_{down}^{i}$	2.5*Jeachtime*

The relevant parameters in the Trans-UTPA experiment are shown in Table 5.

**Table 5:** The relevant parameters in the Trans-UTPA experiment.

**Description**	**Value**
Learning rate of the actor network	0.001
Learning rate of the critic network	0.001
Max clipped value loss	0.2
Entropy regularization coefficient	0.5
Value function update coefficient	0.5
Batch size	512

In the simulation environment, we trained 10,000 times to explore the feasibility of using Trans-UTPA algorithm energy consumption model for UAV group.

**Figure 12:**
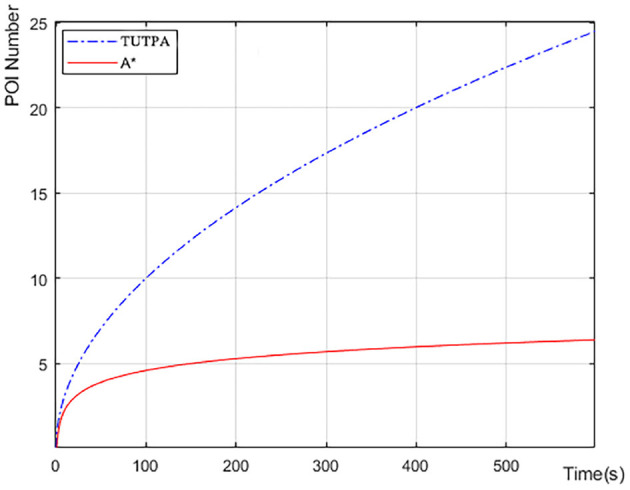
Average number of POI visits for multiple experiments.

Figure 12 shows the comparison of POI visits between Trans-UTPA and A *. The UAV uses Trans-UTPA (Trans-UTPA is abbreviated as TUTPA) algorithm and A * algorithm to complete the mission in the same flight area. From the figure, we can find that the TUTPA algorithm can access more target-POI at the same time, because the TUSCTP algorithm can mobilize the UAV to access more data acquisition areas during the process of going to the network communication area.When accessing the same number of target-POIs, the TUTPA algorithm takes less time than the A * algorithm, which better shows that the TUTPA algorithm can cover more POIs and work efficiently.

**Figure 13:**
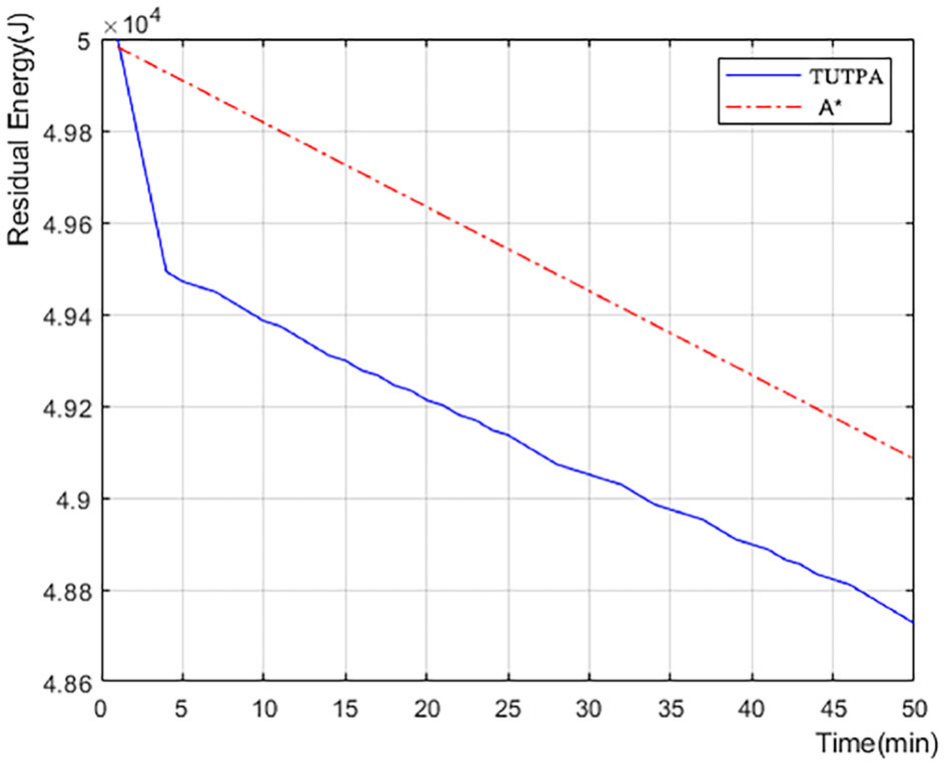
The remaining power of a single UAV after performing data acquisition on target-POI.

Figure 13 we recorded the remaining power of a single UAV starting to perform data acquisition tasks on target-POI. According to the data in Figure 12, the number of target-POI that A * algorithm can access is 6 in 600 s (10 min), and the number of target-POI that TUTPA algorithm can access is 25, which is about 4 times that of A * algorithm. However, in this process, TUTPA consumes less energy than A * in a target-POI area on average. It is concluded that the energy consumption of TUTPA algorithm has certain advantages and can achieve energy saving.

## 6. Conclusion

With the coming of 6G network, UAVs will provide more and more help in emergency disaster relief. We consider the distribution of the rescue area, the type of mission, and the flight characteristics of the UAV. Firstly, according to the distribution of the crowd, the PSO algorithm is used to cluster the target-POI of the task area, and the neural collaborative filtering algorithm is used to prioritize the target-POI. Then we design a Trans-UTPA algorithm and introduce Transformer mechanism to sequence modeling. The UAV completes flight movements (horizontal flight, ascent and descent) and emergency missions (data acquisition and networked communications) in a three-dimensional space, sharing information about the global UAV. The multi-UAVs cooperative flight constraints and multi-UAVs green energy consumption calculation model are designed. The multi-agent reinforcement learning is used to design the flight route according to the maximum global reward. The experimental results show that the Trans-UTPA algorithm makes the success rate of each UAV reaching the target position, the number of collisions and the average reward performance of the algorithm further improve than the USCTP algorithm. Among them, the average reward algorithm exceeds USCTP algorithm 13%, the number of collisions reduced by 60%. Compared with the heuristic algorithm, it can cover more target-POI, and has less energy consumption than the heuristic algorithm. There are still some defects in the algorithm, and there is a lack of three-dimensional actual environment simulation experiments. In the future, we will continue to study along this point.

## Data availability statement

The datasets presented in this study can be found in online repositories. The names of the repository/repositories and accession number(s) can be found in the article/supplementary material.

## Author contributions

JL, SC, XL, RY, and XW conceived the idea of the study and interpreted the results. JL, XL, and SC analyzed the data. SC wrote the paper. All authors discussed the results and revised the manuscript. All authors contributed to the article and approved the submitted version.
